# The Dig It Check It model

**DOI:** 10.1016/j.dib.2018.08.131

**Published:** 2018-08-31

**Authors:** Amy Mosig Way, Amy Tabrett

**Affiliations:** The University of Sydney, Australia

## Abstract

This data article describes how the Dig It Check It (DICI) model operates. The purpose of the DICI model is to assess the effectiveness of a specified subsurface sampling program in the detection of archaeological sites (Way and Tabrett, in press) [1]. Specifically, the aim of the model is to determine the inherent biases of the specified sampling program, i.e. what is the probability that sites of a certain size, density and density-distribution will be, or were, missed by the sampling program. A simulation is run which randomly places sites, the characteristics of which can be varied between runs, in a sampling area with the specified test-pit layout to determine the probability of overlap between the test-pits and sites with the selected characteristics. When overlap does occur, the site is recorded as intersected. The model then calculates the site density at that point and compares it with the test-pit size to determine whether the density is great enough for detection to also occur.

**Specifications table**

**Table:** 

Subject area	Archaeology
More specific subject area	Sampling effectiveness
Type of data	NetLogo Model
How data was acquired	NetLogo Model
Data format	Code
Experimental factors	NA
Experimental features	There are three entities in the model: survey area, sites and test-pits. The survey area variables are determined by a user-imported shapefile. The variables of the sites are diameter, density and density-distribution. The variables of the test-pits are length, width and location.
Data source location	NA
Data accessibility	The model is available at http://www.digittools.net/
Related research article	A.M. Way and A. Tabrett, A new simulation tool for the assessment of subsurface testing: Dig It Check It, J. Archaeol. Sci. Rep. 21 (Oct), 2018, 71-75

**Value of the data**

• Dig It, Check It is the first model to be presented which employs simulation to assess the effectiveness of irregular, real-world subsurface sampling programs in the detection of concentrations of artefacts.
• One of the principal points of difference between this new model and previous work is that this tool allows real-world and irregular test-pit layouts to be analysed through the importation of a GIS shapefile.
• This means that a sampling strategy which includes test-pits of varying sizes and intervals can be assessed, as can survey areas of non-rectangular shape.
• This allows the archaeologist to easily conduct a statistically rigorous bias assessment of any subsurface testing program.

## 1 Data

The data presented here is a NetLogo [2] model which is available online at http://www.digittools.net/ [1]. The model has three entities: survey area, test-pits and sites. The aim of the model is to analyse the relationship between the test-pits and the artefact sites. Specifically, the aim is to determine what percentage of sites with the specified properties are detected by the test-pits in that survey area. The site variables can be adjusted between simulation runs to determine the relationship between the sampling strategy and sites with different properties.

## 2 Model design and entities

There are three entities in the model: survey area, sites and test-pits. A simulation is run which randomly places sites in a survey area which contains the specified test-pits to determine the probability of overlap between the test-pits and sites. When overlap does occur, the site is recorded as intersected. The model then calculates the site density at that point and compares it with the test-pit size to determine whether the density is great enough for detection to also occur.

### 2.1 Survey area

The survey area is loaded through the importation of two shapefiles via the interface. The survey area boundary file sets the world envelope and scales and resizes the display of the world/NetLogo screen. The second shapefile draws the survey area boundary and codes the area within the boundary as ‘surveyed’. The following pseudo-code describes this process:

**Table:** 

*to load-survey-area-boundary*
*clear-all*
*load the world shapefile*
*let the envelope be set by the world file*
*load the survey-area-boundary shapefile*
*draw the survey area boundary*
*code the patches within the survey area as ‘surveyed’*
*end*

### 2.2 Test-pits

Pits have the variables: ID, *x* coordinate, y coordinate, length, width, area, artifact-density, number of sites intersected and number of sites detected. The ID, *x* coordinate, *y* coordinate, length and width are imported with the shapefile, and the area is then calculated from the length and width variables.

Each pit consists of a pit-set, which includes a single seed-pit, which is imported as a point during the importation of the shapefile, and additional pit-squares. The initial seed-pits create a pit-set of multiple pit-squares, which together define the extent of each test-pit. This allows test-pits of different sizes to be imported and constructed. These pit-squares have the same ID as the seed-pit, and the seed-pit and pit-squares keep a tally of how many sites the test-pit intersects and detects. This allows sites which partially overlap a pit-set to be recorded as intersected/detected by the test-pit as a single unit. The following pseudo-code describes this process:

**Table:** 

*To add-test-pits*
*Import the test-pit shapefile and add a seed-pit at each point*
*Set the x-coordinate and y-coordinate from the shapefile data*
*Set the pit-width and pit-length (these are set by the width and length variables in the attribute table of the.shp. These variables don’t affect the display of the seed-pit, but determine how may pit-squares will be added in the next step to define the extent of the total test-pit)*
*Set the pit ID (imported from the.shp)*
*Set pit area (length × width) (the seed-pit then creates the rest of the test-pit by adding the required number of pit-squares)*
*Set size and color*
*end*

## 3 Sites

The sites consist of concenrations of artefacts. The site-properties are established during the initialisation on the interface with the variables ‘average-density’, ‘site-diameter’ and ‘density-distribution’. These properties can be adjusted between runs.

The size of the site is determined by the variable ‘site-diameter’. The density of each point within the site is calculated according to the average-density and the density-distribution. The density distributions which can be selected are: uniform, linear, cosinusoidal, and the Lake George Regression (LGR) curve. The density distributions are described below.

In the uniform density distribution all points within the site will have the same density as the average density. If the density distribution is set to a linear, then a regular density decay occurs. The centre of the site will have the highest density, which is set at 3.0 times the average density. Decay then proceeds linearly to zero at the margin of the site. This density distribution is calculated with the following equation:

setartifact-density((average-density*3)−(r/(site-diameter/2)*(average-density*3)))

where *r* = the distance of the point of intersection from the centre of the site.

In the cosinusoidal distribution, the centre of the site has a maximum density of 3.36 times the average density. Density decay occurs slowly in the central area of the site and then increases towards the margin, until it falls to zero at the perimeter. This creates a site with a large central area of high density, a medium density through the centre of the site, and a low density margin. This distribution is determined by the equation:

setartifact-density((average-density*3.36)/2)+(((average-density*3.36)/2)*cos(((2*180)/site-diameter)*r)))

In the LGR density distribution the centre of the site has a maximum density of 6.66 times the average density. This curve produces a small central area of high density, a steep decay through the centre of the site, and a large marginal area of low density, which falls to zero at the perimeter of the site. This distribution is determined by the equation:

$$set\;artifact-density\;\left((average-density*6.66)*\left(1-\left(y-score^{2}\right)\right)\right)$$

where *y*-score is the standard deviation of the site at that point as determined by the radius.

The sites are added to the model by randomly placing a seed-site within the surveyed area. The seed-site then builds the rest of the site according to its diameter and density-distribution.

In some situations, artifacts are not distributed in these ways, but are clustered. Similar to Kintigh׳s [3] programs, DICI allows the archaeologist to model clustered distributions by switching ‘Clustering?’ to on. The archaeologist then enters a clustering parameter k, a number between 0 and 1 where smaller values correspond to more clustered artifact distributions. Instead of using the Poisson distribution to model the probability of finding at least one artifact in a test unit, for a clustered distribution the negative binomial distribution is used [3]:

$$p\left(\mathrm{finding}\geq 1\;\mathrm{artefact}\right)={\left(\frac{k}{k+d}\right)}^{k}$$

where *k* = clustering parameter and *d* = expected artifact density in test-pit

## 4 Relationship between entities

The survey area and the test-pits are now loaded and drawn, and the characteristics of the sties have been established on the user-interface. The simulation is now ready to run. During the simulation the sites are added to random locations within the survey area. The number of sites added is determined by the user through the variable ‘trial-runs’, which is set by default to 1000. If a site overlaps a single test-pit, the intersection tally for that test-pit increases by 1. During this calculation, all the pit-squares in a pit-set ask their fellow pit-squares if they intersected any sites in that run. This allows sites which only partially overlap a test-pit to be tallied as intersected by the test-pit as a single unit.

The detection calculation occurs in a similar manner, however a detection threshold of 1 is set here. If this site overlaps a test-pit, the artifact density of the site at the point of overlap is transferred to the pit-square. All the pit-squares then calculate the total pit density by adding together the artifact-densities of each pit-square in the pit-set and then dividing that figure by the total pit area. If the total pit density is greater than 1, the site is recorded as detected, and the site detection tally is increased by 1. The pseudo-code for this process is as follows:

**Table:** 

*to run-simulation*
*add site (this randomly adds the site to the survey area)*
*intersect-sites (if there is a test-pit where the site is located, then an intersection is recorded. This number increases by one as subsequent sites are intersected)*
*detect-sites (if there is a test-pit where the site is located, and the total pit density/pit area is greater than or equal to one, then a detection is recorded. This number increases by one as subsequent sites are detected)*
*clear-sites-display (this removes the display of the site before the next site is added)*
*stop after the number of trial runs is reached*
*end*

**Fig. 1:**
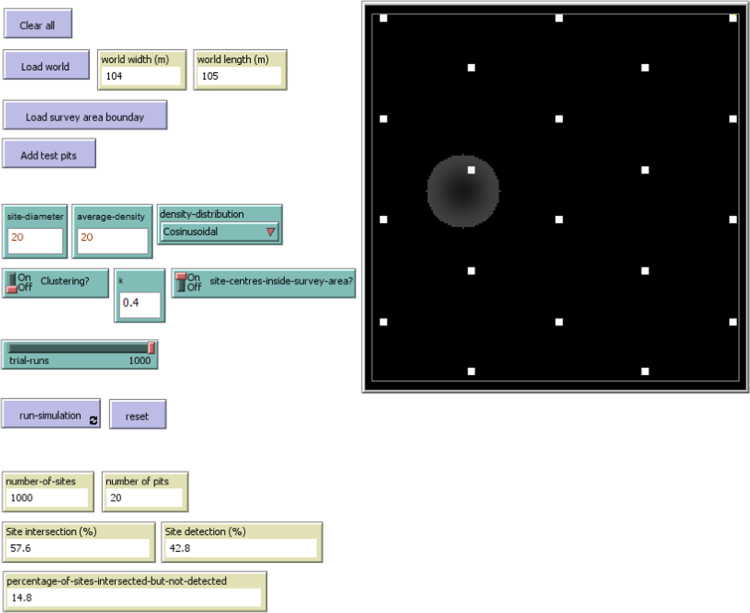
Model interface at end of run.

The simulation runs until the number of trial runs is reached. At this point, the monitors can be inspected (see Fig. 1). The first two monitors display the number of test-pits and the number of sites loaded during the setup phase and during the simulation. The next three monitors display the percentage of sites detected, the percentage of sites intersected, and the percentage of sites intersected, but not detected. The pseudo-code for these three monitors is as follows:

**Table:** 

*to-report percentage of sites detected*
report (number of sites detected)/(total number of sites) * 100
*end*
*to-report percentage of sites intersected*
report (number of sites intersected)/(total number of sites) * 100
*end*
*to-report percentage of sites intersected but not detected*
report (number of sites intersected but not detected)/(total number of sites) * 100
*end*

## 5 Underlying principles

The concepts of intersection probability and detection probability, which were first introduced by Krakker et al. [4] underlie the model׳s design. Intersection probability is determined by the probability that a test-pit and site will overlap, while the probability that a site will also be detected is determined by the relationship between the density of the site at the point of intersection and the test-pit size. Krakker et al. relied upon the Poisson sampling distribution to determine the probability that the test-pit would contain at least one artifact. With this assumed distribution, they found that the probability of finding at least one artifact in a test-pit could be calculated as follows:

$$p=1–e^{-\mathrm{ad}}$$

where *p* is the probability of finding one or more artifacts, *a* is the area of the test-pit and *d* is the artifact density [4].

Randomness/stochasticity is also necessary for modelling the presence of artifacts within an intersected test-pit. The Poisson distribution (see equation above) is used to calculate the probability of finding at least one artifact in a test-pit based on the expected number of artifacts at the point of intersection [3]. Stochasticiy is then introduced to simulate the random positioning of the artifacts within the test-pit. At this point a random number is selected between 0 and 1 and if this number is less than or equal to the probability of detection, the test-pit detects an artifact, and therefore a site.

Randomness/stochasticity is also required when a site is generated. During the simulation a number of trials are run. During each trial a site is randomly placed within the survey area. In this way stochasticity is used to simulate the unknown nature of the location of buried sites in the landscape. If detection and/or intersection occur, this is tallied. Each trial is independent of the others.

The purpose of the model is to determine the probability that a site will be detected or intersected by a particular sampling strategy.

## 6 Limitations

The minimum test-pit size is hard-coded into the model to be 0.25 m^2^ to prevent the model from running too slowly. As such, the test-pits are restricted by this format and can only consist of combinations of 0.25 m^2^ squares. They cannot be circular.

The base unit for the construction of the sites is the NetLogo ‘turtle’, which can be combined to produce a unit of any shape. However, the sites constructed in this model are circular. In this paper ‘site’ means an artifact concentration, and after Krakker et al. [4] and Kintigh [3] sites are assumed to be circular in shape.
